# A Dissipative Particle Dynamics Study of Flow Behaviors in Ultra High Molecular Weight Polyethylene/Polyamide 6 Blends Based on Souza-Martins Method

**DOI:** 10.3390/polym11081275

**Published:** 2019-07-31

**Authors:** Junxia Wang, Ping Li, Changlin Cao, Shijie Ren, Dingshan Yu

**Affiliations:** 1 Key Laboratory for Polymeric Composite and Functional Materials of Ministry of Education and Key Laboratory of High Performance Polymer-based Composites of Guangdong Province, School of Chemistry, Sun Yat-Sen University, Guangzhou 510275, China; 2 State Key Laboratory of Polymer Materials Engineering, Sichuan University, Chengdu 610065, China

**Keywords:** dissipative particle dynamics, flow behaviors, ultra high molecular weight polyethylene, Souza-Martins method

## Abstract

This paper presents our study on the use of dissipative particle dynamics (DPD) simulations to discover the flow behavior in ultra high molecular weight polyethylene/polyamide 6 (UHMWPE/PA6) blends associated with extensional-shear coupled flow, based on the Souza-Martins method, for the first time. By way of simulations, we aimed at investigating the mesoscopic morphology and alignment behavior in response to extensional-shear coupled flow, in comparison with simple shear flow and simple extensional flow. Our results reveal that the aggregation of polymers is noticeable under zero flow, as expected. Within the considered range of extensional-shear coupled rates, the morphology transforms from micelle-like clusters to a chain-like network structure by increasing coupled rates from 0.01 to 2.0. Furthermore, it shows a linear distribution along the flow direction at a high coupled rate. It can be concluded that the flow behaviors in UHMWPE/PA6 blends are significantly impacted by extensional-shear coupled rates. The orientation behavior induced by extensional-shear coupled flow is more obvious than shear flow, even though flow variations and mass fractions yield less effects on the distribution behaviors of UHMWPE/PA6 blends. The DPD results are verified by mean square displacement (MSD) as a function of simulation time and relative concentration distribution along Z direction.

## 1. Introduction

It is well-established that morphology and interfacial behavior play key roles in the rheological properties, when blending or mixing immiscible polymer melts, and thus affects the final mechanical properties of the blends [1,2]. Hence, it is vital to elucidate the connection between applied flow fields and morphology evolutions for optimization of the processing, and therefore, the resulting properties of blends, taking interfacial tension into account [3,4].Various thermodynamic variables change continuously over this interfacial region and the thickness of this interfacial region is associated with the finite range of molecular interaction. The finite interaction range is characterized by the free energy of the system, which is dependent not only on the local composition but also on the composition of the immediate environment [5]. For immiscible polymer pairs with infinite molecular weights, interfacial tension can be calculated according to the Flory–Huggins interaction parameter (χ) between the corresponding monomers; e.g., A and B, given the assumption of complete immiscibility [4,6]. Mixing performance is related to the energy to mix, reflected in overall pressure drop for all designs [7]. Small chains cluster at the interface, resulting in the reduction of the interfacial tension and the Gibbs free energy of the system [4,8].

Factually, for ultrahigh molecular weight polyethylene (UHMWPE) samples prepared by eccentric rotor extrusion [9,10,11], the oriented structure has been experimentally observed and evidenced by Raman spectra [12]. However, it is plagued by experimental difficulty in studying the dynamic process of chain conformation. Compared with experimental study, the unique advantage of computational simulation, e.g., coarse-grained molecular dynamics (CGMD) and dissipative particle dynamics (DPD) simulation, lies in directly recording, tracking and observing the dynamic evolution process in real time, offering a complement to experimental studies.CGMD and DPD are coarse-grained simulation techniques based on molecular dynamics (MD), where each beadrepresents several atoms or repeat units in MD [13,14]. Even though they are employed for studying systemswithlarger lengths and time scales than classical MD dynamics [15,16,17], only in DPD simulation the interfacial properties can be taken into consideration. For engineering applications, mixing UHMWPE with polyamide 6 (PA6) is an effective and convenient route to reduce the melt viscosity.In our earlier report [18], great efforts have been made to compare the complex phase morphology and alignment evolution of the immiscible UHMWPE/PA6 blends in response to extensional and shear flow by means ofDPD simulation, in which the extensional flow and shear flow were separately imposed. During the actual process of eccentric rotor extrusion, the polymer melts encounter an extensional and a shear deformation at the same time; that is, they undergo extensional-shear coupled flow. The extensional-shear coupled flow can be realized by adjusting the magnitude of extensional loading in Z direction and X-Y shear loading by utilizing finite element simulation [19]. Finite element method is a typical success of continuum mechanics in predicting materials’ response and failure at macroscopic level. To extend the concepts of continuum mechanics, i.e., stress and strain, to the nano-scale for exploring the microscopic behavior, attracts increasing attention [20,21].Inspired by this, we make an attempt to construct the extensional–shear coupled flow in DPD simulation, for the first time. In Reference [18], based on the Souza–Martins method [22], the extensional flow was driven by an external pressure along the length L_z_ of the simulation box. Simultaneously, the Lees–Edwards boundary condition [23] was first proposed in 1972 for achieving a linear velocity profile, and then it was used for producing a shear flow in Y direction with the velocity gradient in X direction [24]. Likewise, we can establish shear flow utilizing the Souza-Martins method, similar to the extensional flow, in which external pressure along the lengths L_x_ and L_y_ of the simulation box are imposed on each bead with identical magnitude, respectively. In this case, the extensional flow and shear flow was simultaneously imposed on each bead, being consistent with the actual process of eccentric rotor extrusion. The Souza-Martins method is found to function more effectively under an extensional–shear coupled flow field, than Lees–Edwards boundary conditions. Hence, in this contribution, we present a study on the use of DPD simulations to investigate the flow behavior in UHMWPE/PA6 blends subjected to extensional–shear coupled flow by varying extensional–shear coupled rates, in comparison with simple shear flow and simple extensional flow, based on the Souza-Martins method.

## 2. Theoretical Background and Simulation Details

### 2.1. The Interfacial Properties between Immiscible Polymers

The interfacial tension ($\gamma_{\infty }$) in relation to the χ is demonstrated in Equation (1) [6,8].

(1) $$\gamma_{\infty }=\frac{k_{B}T}{a^{2}}{\left(\chi /6\right)}^{1/2}$$

where a is the statistical segment (monomer) length, $k_{B}$ is the Boltzmann constant and *T* is the absolute temperature.

The χ of UHMWPE and PA6 pairs can be estimated from the change in energy during mixing per unit volume using Equation (2) [25] and Equation (3) [26].

(2) $$\chi =\left(\frac{\Delta E_{\mathrm{mix}}}{RT\phi_{A}\phi_{B}}\right)V_{m}$$

(3) $$\Delta E_{\mathrm{mix}}=\phi_{A}{\left(\frac{E_{coh}}{V}\right)}_{A}+\phi_{B}{\left(\frac{E_{coh}}{V}\right)}_{B}-{\left(\frac{E_{coh}}{V}\right)}_{mix}$$

where $\phi_{i}$ is the volume fraction and an implicit condition relating to this equation is that the lattice is filled completely, namely, $\phi_{A}$ + $\phi_{B}$ = 1. $V_{m}$ and V are the average molar volume of the beads and unit volume of polymer system, respectively. $E_{coh}$ is the calculated cohesive energy, which can be directly calculated by MD simulation as depicted in detail in our earlier work [18].

Then the repulsive parameter $\alpha_{\mathrm{ij}}$ between different types of beads is calculated according toχ, see Equation (4). When i equals j, the repulsive parameter $\alpha_{\mathrm{ii}}$ between two identical type beads can be obtained using Equation (5):

(4) $$\alpha_{\mathrm{ij}}\approx \alpha_{\mathrm{ii}}+3.27\chi_{ij}$$

(5) $$\alpha_{\mathrm{ii}}=\frac{75k_{B}T}{\rho }$$

where the bead number-density, ρ, is chosen as 3 and *k_B_T* is the conservative interaction potential chosen as 1.

### 2.2. Construction Basis in Flow Field

**Figure 1:**
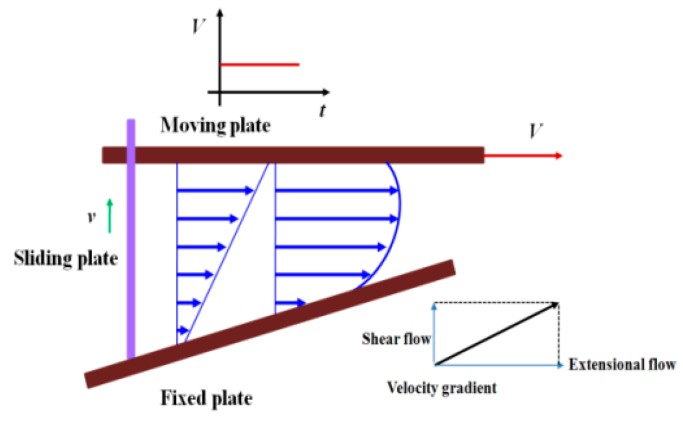
Schematic diagram of extensional–shear coupled flow.

As mentioned above, in the actual process of eccentric rotor extrusion, the polymer melts undergo a combination of extensional deformation and shear deformation; see Figure 1.

For simple shear flow, the velocity gradient $\dot{\gamma }(t)$ is given by Equation (6) [27]:

(6) $$\dot{\gamma }(t)=\dot{\gamma }(t)\left(\begin{array}{ccc} 0 & 1 & 0\\ 1 & 0 & 0\\ 0 & 0 & 0 \end{array}\right)$$

For simple extensional flow, the velocity gradient $\dot{\varepsilon }(t)$ can be expressed as [28]:

(7) $$\dot{\varepsilon }(t)=\dot{\varepsilon }(t)\left(\begin{array}{ccc} -1 & 0 & 0\\ 0 & -1 & 0\\ 0 & 0 & 2 \end{array}\right)$$

The velocity gradient ∇u for a combination of shear flow and extensional flowhas the following equation [29]:

(8) $$\nabla u=\left(\begin{array}{ccc} -\dot{\varepsilon } & \dot{\gamma } & 0\\ \dot{\gamma } & -\dot{\varepsilon } & 0\\ 0 & 0 & 2\dot{\varepsilon } \end{array}\right)$$

In Materials Studio, pressure is another basic thermodynamic variable that defined as the force per unit area and the general form is shown in Equation (9):

(9) $$P=\left(\begin{array}{ccc} P_{xx} & P_{xy} & P_{xz}\\ P_{yx} & P_{yy} & P_{yz}\\ P_{zx} & P_{zy} & P_{zz} \end{array}\right)$$

Each element of the tensor is the force applied on the surface of an infinitesimal cubic volume with edges parallel to the *x*, *y*, and *z* axes. The first subscript represents the direction of the normal to the plane and the second subscript refers to the component of the force.

In an isotropic simulation, the off-diagonal elements are zero and the diagonal elements are equal, where the forces are the same in all directions and there is no viscous force, see Equation (10):

(10) $$P=p\left(\begin{array}{ccc} 1 & 0 & 0\\ 0 & 1 & 0\\ 0 & 0 & 1 \end{array}\right)=pI$$

where the scalar quantity, p, is the equivalent hydrostatic pressure.

Sometimes, especially in materials science studies, the diagonal elements are acknowledged as the tensile or normal stress and the off-diagonal elements are known as the shear stress. Both pressure and stress are input and reported in GPa in Materials Studio.

Consequently, in Materials Studio, simple shear flow ($P_{S}$), simple extensional flow ($P_{E}$) and extensional–shear coupled flow ($P_{E-S}$) can be described as Equations (11)–(13), respectively, in which the equivalent hydrostatic pressure *p* is zero.

(11) $$P_{S}=\left(\begin{array}{ccc} P_{xx} & P_{xy} & P_{xz}\\ P_{yx} & P_{yy} & P_{yz}\\ P_{zx} & P_{zy} & P_{zz} \end{array}\right)=\left(\begin{array}{ccc} 0 & P_{xy} & 0\\ P_{yx} & 0 & 0\\ 0 & 0 & 0 \end{array}\right)$$

(12) $$P_{E}=\left(\begin{array}{ccc} P_{xx} & P_{xy} & P_{xz}\\ P_{yx} & P_{yy} & P_{yz}\\ P_{zx} & P_{zy} & P_{zz} \end{array}\right)=\left(\begin{array}{ccc} P_{xx} & 0 & 0\\ 0 & P_{yy} & 0\\ 0 & 0 & P_{zz} \end{array}\right)=\left(\begin{array}{ccc} P_{xx} & 0 & 0\\ 0 & P_{xx} & 0\\ 0 & 0 & -2P_{xx} \end{array}\right)$$

(13) $$P_{E-S}=\left(\begin{array}{ccc} P_{xx} & P_{xy} & P_{xz}\\ P_{yx} & P_{yy} & P_{yz}\\ P_{zx} & P_{zy} & P_{zz} \end{array}\right)=\left(\begin{array}{ccc} P_{xx} & P_{xy} & 0\\ P_{yx} & P_{xx} & 0\\ 0 & 0 & -2P_{xx} \end{array}\right)$$

### 2.3. Simulation Details

On the basis of Equations (2)–(5), the repulsive parameter α_ij_ between UHMPWPE and PA6 is 38.18, 52.08 and 57.83 in UHMWPE/PA6 blend with mass fractions of 70/30, 50/50, 30/70, respectively. More details can be found in our earlier work [18].The simulation was carried out using the Mesocite module in Accelrys Materials Studio software. The extensional–shear coupled flow is generated by imposing a force on each bead in three directions, in which the pressure and stress are controlled by Souza-Martins method, according to Equations (11)–(13).Initially, the simulation was accomplished in a cubic box with a bead mass of 685 amu and length scale of 12.8 Å in reduced DPD unit. The coarse-grained model consists of 42,521 beads (11,596 beads for every UHMWPE chain and 51beads for every PA6 chain).Initial condition for the simulation: DPD simulation of UHMWPE/PA6 blends were performed under zero flow after 1.2 × 10^5^ steps geometry optimization and 1.0 × 10^5^ steps dynamic simulation, with a time step of 1fs in reduce DPD unit to ensure that the system reached a complete equilibration, judging from the individual stable stage in the time evolution of pressure and total potential energy of the system (see Figure S1).

## 3. Results and Discussion

In this work, Souza-Martins method is performed for driving the shear flow field, instead of Lees–Edwards boundary conditions. The Lees–Edwards boundary conditions have been commonly applied in MD to generate a shear flow with a constant shear rate and extended into DPD to eliminate the wall effect, in which the periodic boundary conditions are applied for the whole domain, including the top and bottom boundaries. A constant force was applied on all beads to generate simple shear flow, and the shear rates varied from 0.01 to 2, where $\dot{\gamma }=0.01–2$, $\dot{\varepsilon }=0$ (*P_yx_* = *P_xy_* = 1.0 × 10^−5^–2.0 × 10^−3^ GPa and *P_xx_* = *P_yy_* = −1/2*P_zz_* = 0 GPa).

**Figure 2:**
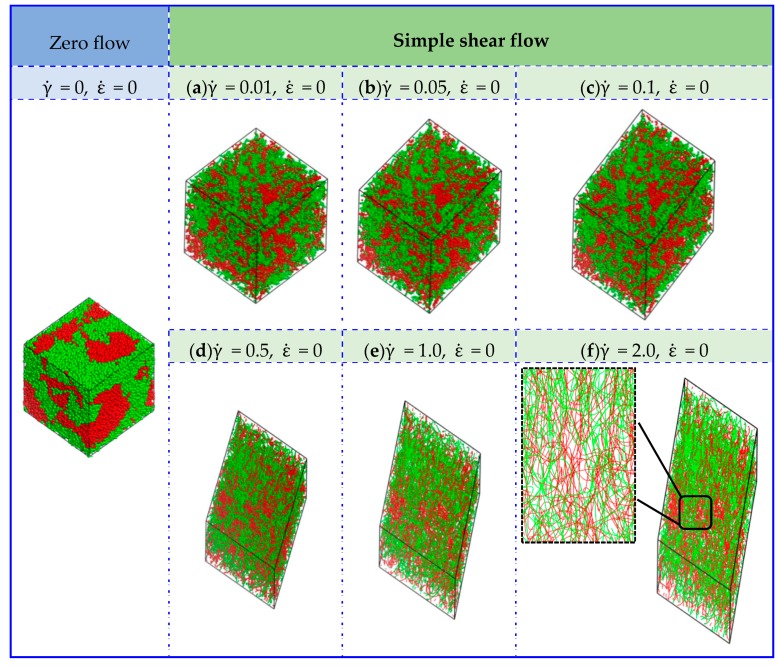
Conformation of UHMWPE/PA6 blends at50/50 mass fractionsunder different shear rates. The red and blue beads represent the PA6 and UHMWPE polymers, respectively. (**f**) is displayed in line style and the others are presented in dot-line style.

Figure 2 displays the equilibrium structures of UHMWPE/PA6 blends to 50/50 mass fractions with respect to zero flow and simple shear flow. The red and blue beads represent the PA6 and PE polymers, respectively. Self-assembly and random distribution of UHMWPE and PA6 under zero flow can be observed as expected. In the simple shear flow, UHMWPE/PA6 blends show different morphologies as well as the distribution, depending on the strength of shear flow. At a low shear rate of $\dot{\gamma }$ = 0.01, the PA6 polymers self-assemble into small aggregates and form micelle-like clusters, in which the size of the agglomerated polymers decreases and the number increases. Nevertheless, micelle-like clustersdisappear at large values of shear rates (i.e., $\dot{\gamma }$ = 2.0), and then chain-like morphology occurs and the molecular orientation is parallel to the flow direction, finally giving a linear distribution and a good compatibility between UHMWPE and PA6molecules.

**Figure 3:**
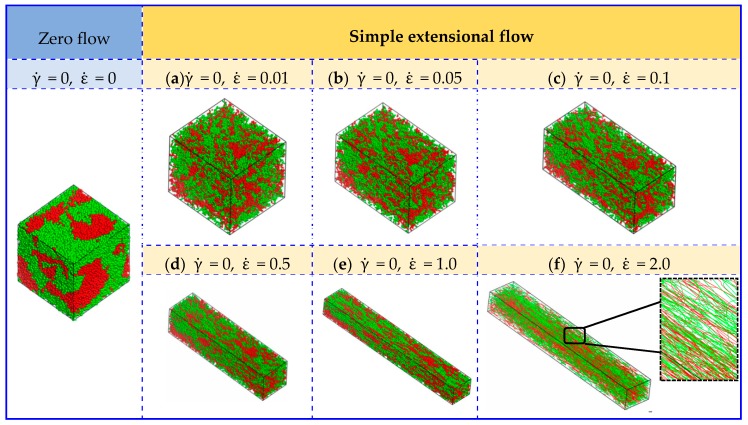
Conformationof UHMWPE/PA6 blends at 50/50 mass fractions under different extensional rates. The red and blue beads represent the PA6 and UHMWPE polymers, respectively. (**f**) is displayed in line style and the others are presented in dot-line style.

In this subsection, the extensional flow also has been taken into consideration and it brings an significant effect on the morphological behaviors of UHMWPE/PA6 blends, under a range of extensional rates, in which $\dot{\gamma }$ = 0, $\dot{\varepsilon }$ = 0.01–2 (*P_yx_* = *P_xy_* = 0 GPa and *P_xx_* = *P_yy_ =* −1/2*P_zz_* = −1.0 × 10^−5^–2.0 × 10^−3^ GPa). Figure 3 presents the typical equilibrium structures as a function of extensional rates $\dot{\varepsilon }$. It shows almost the same structural evolutions as the condition of shear flow when the extensional rate is low (i.e., $\dot{\varepsilon }$ = 0.05). With an increased $\dot{\varepsilon }$ of 1.0, the PE and PA6 molecules in the blended system are elongated and exhibit strong tendency to parallel align along the flow direction. By increasing $\dot{\varepsilon }$ to 2.0, the micelle-like structures finally show linear distribution and transforms to chain-like network structures.

**Figure 4:**
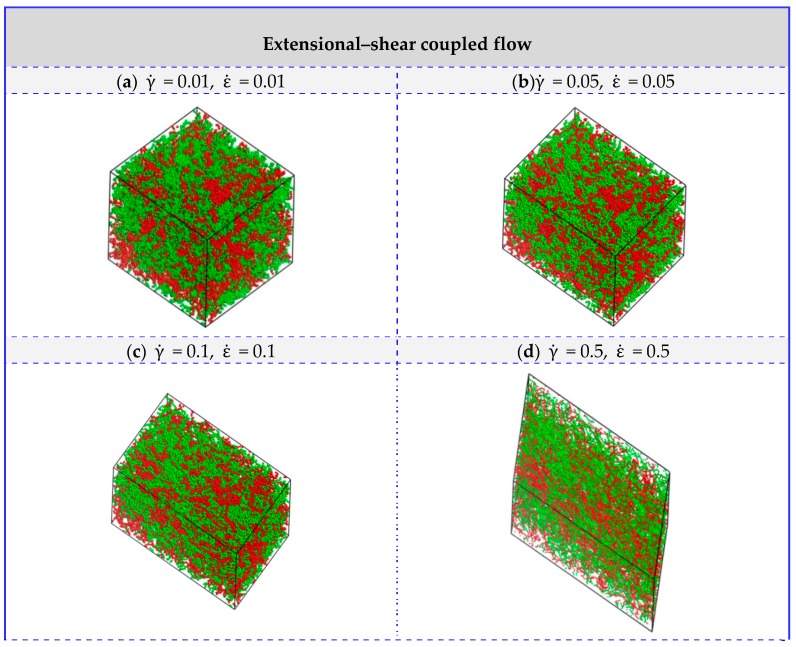

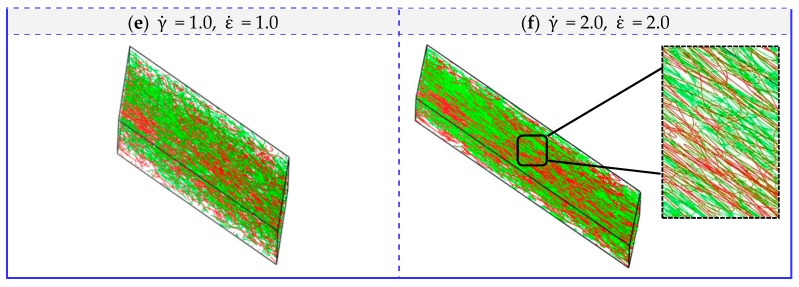
Morphologies of UHMWPE/PA6 blends of 50/50 mass fractions under extensional–shear coupled flow. The red and blue beads represent the PA6 and UHMWPE polymers, respectively. (**e**,**f**) are displayed in line style and the others are presented in dot-line style.

To further investigate the effect of extensional–shear coupled rate on the morphology and distribution in the blended UHMWPE and PA6, a combination of the above simple shear flow and extensional flow was then applied. Figure 4 is a plot of the mesoscopicmorphology of UHMWPE/PA6 blends responding to an extensional–shear coupled flow, with varying levels of shear rates and extensional rates, such as $\dot{\gamma }$ = 0.01–2, and $\dot{\varepsilon }$ = 0.01–2 ($\dot{\gamma }=\dot{\varepsilon }$). As seen in Figure 4, the equilibrated morphologies and distributions of the polymers are very different in the range of 0.01–2.0. The micelle-like clusters are noticeable at a low $\dot{\gamma }$ and $\dot{\varepsilon }$ (from 0.01 to 0.1) because the flow is also not strong enough to disturb the microstructure and the morphology remainsalmost unchanged. With further increasing $\dot{\gamma }$ and $\dot{\varepsilon }$, the molecular chains are highly oriented. The distribution of PE and PA molecules are uniform by changing coupled rates, maintaining a good compatibility between PE and PA6. Based on the analysis above, it can be concluded that the coupled rate applied to the UHMWPE/PA6 blends more significantly affect the morphologies than the distribution of the polymers, which should be taken into account in the preparation of UHMWPE/PA6 blends. It is worth noting thatfrom the enlarged view of Figure 2f and Figure 4f, the orientation of UHMWPE and PA chains induced by extensional–shear coupled flow is more obvious than shear flow, which is in accordance withexperimental Raman spectra results in Reference [12].

**Figure 5:**
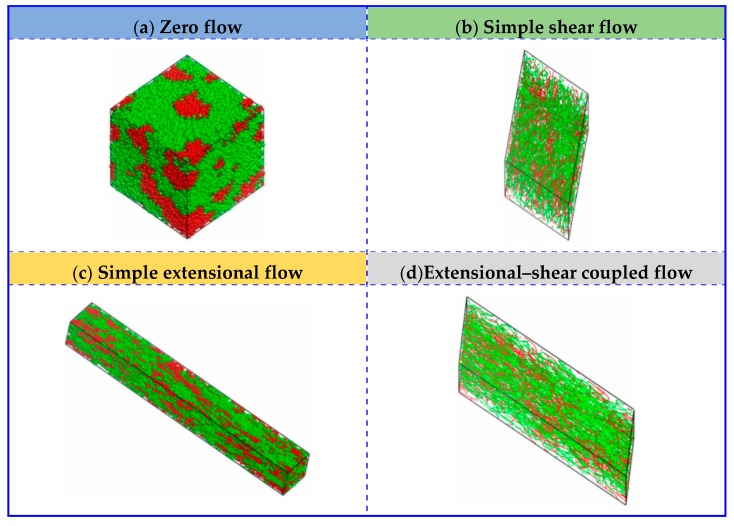
Comparison of UHMWPE/PA6 blends of 70/30 mass fractions under zero flow, simple shear flow, simple extensional flow and extensional–shear coupled flow.(**b**,**d**) are displayed in line style and the others are presented in dot-line style.

**Figure 6:**
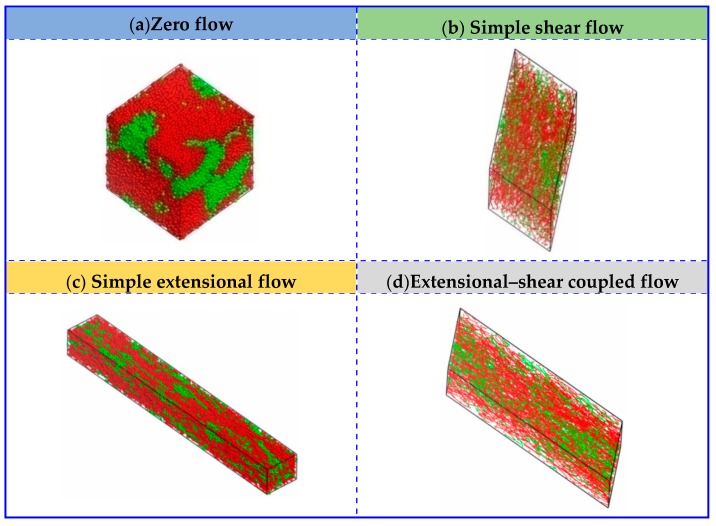
Comparison of UHMWPE/PA6 blends of 30/70 mass fractions under zero flow, simple shear flow, simple extensional flow and extensional–shear coupled flow.(**b**,**d**) are displayed in line style and the others are presented in dot-line style.

In this section, the blends of UHMWPE and PA6 at different UHMWPE/PA6 mass fractions (70/30, 50/50 and 30/70) are considered. Figure 5 and Figure 6 depict the comparison of flow behaviors (zero flow, simple shear flow, simple extensional flow and extensional–shear coupled flow) for systems of UHMWPE/PA6 blends of 70/30 mass fractions and 30/70 mass fractions, respectively. Based on the results above, along with the consideration of the shear rates and extensional rates, we chose the shear rate and extensional rate as 1.0, in our simulations as presented below. In response to simple extensional flow, the micelle-like cluster is elongated along the flow direction. By applying simple shear flow and extensional–shear coupled flow, they enable chain-like network morphologies and uniform linear distribution, even though mass fraction varies. These results agree well with the systems of UHMWPE/PA6 blends at 50/50 mass fractions.

**Figure 7:**
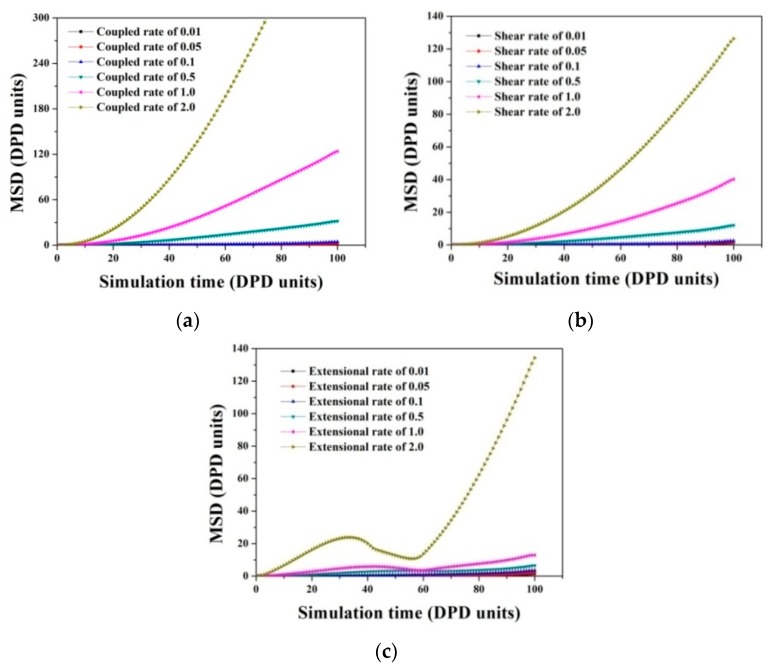
The mean square displacement (MSD) of PA6 at different simulation times corresponding to: (**a**) Extensional–shear coupled flow, (**b**) simple shear flow and (**c**) simple extensional flow at different rates.

**Figure 8:**
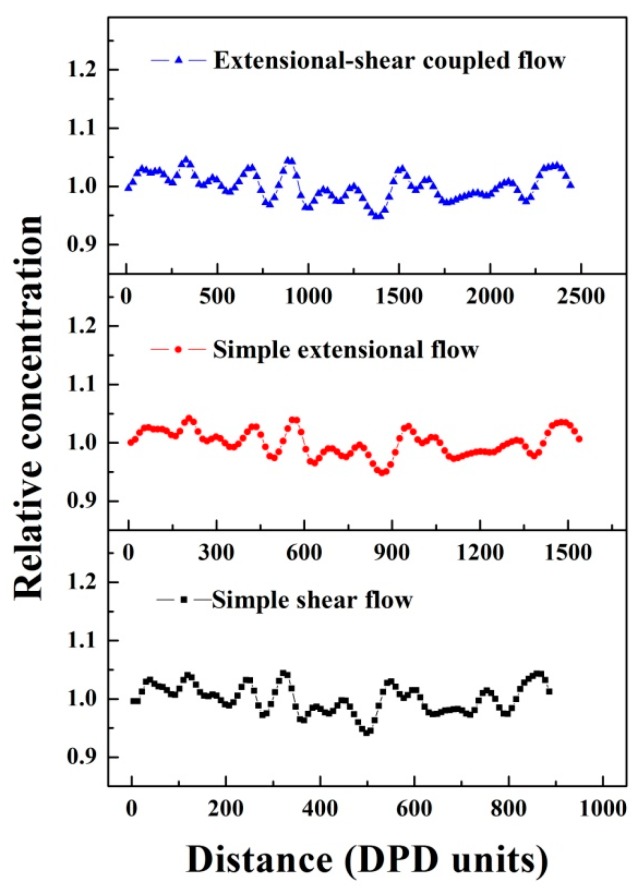
The relative concentration distribution of PA6 along Z direction under simple shear flow, simple extensional flow and extensional–shear coupled flow.

To shed more light onto the morphology and alignment behavior corresponding to extensional–shear coupled flow, we plot the mean square displacement (MSD) of PA6 as a function of simulation time for different coupled rates in Figure 7, as well as shear rates and extensional rates. MSD can be acknowledged as more effective parameter to characterize the average motion velocity of particles compared with the diffusion coefficient and it is calculated by Equation (14) [30,31]:

(14) $$MSD=\langle {\left|\vec{r}(t)-\vec{r}(0)\right|}^{2}\rangle$$

where $\vec{r}(t)$ and $\vec{r}(0)$ refer to the coordinate of a particle at time *t* and 0, respectively. The slope of the MSD-time curve represents the intensity of activity in polymer chains (or beads). We noticed that the slopes of MSD curve in Figure 7a exhibit slight increases upon increasing coupled rates because increasing coupled rates can enhance the random motion velocity of polymer chains, consequently raising the diffusivity of polymer chains, which provides clear proof that the alignment in UHMWPE/PA6 blends are significantly impacted by extensional–shear coupled rates, similarly in the case of shear flow and extensional flow (see Figure 7b,c). Figure 8 displays the relative concentration distribution of PA6 along Z direction. From the number and magnitude of the peak values, the distribution behavior can be evaluated.The fewer peaks and smaller the peaks values, the more uniform the distribution presents in the blends. An almost similar trend of the number and magnitude of peak values in relative concentration distribution curves is observed by comparingextensional–shear coupled flow, simple shear flow and simple extensional flow. This indicates that flow variations yield less effect on the distribution behaviors, in agreement with previous DPD results.

## 4. Conclusions

In this contribution, the Souza-Martins method was adopted in DPD simulation for predicting the flow behaviors in UHMWPE/PA6 blends, in which the effects of simple shear flow, simple extensional flow and extensional–shear coupled flow by varying shear rates, extensional rates and extensional–shear coupled rates, respectively, on the morphologies and distributionwere investigated for the first time. It was found that the flow variations and mass fractionsyielded lesser effects on the distribution behaviors. Nevertheless, the orientation behavior induced by extensional–shear coupled flow was more obvious than shear flow, in accordance with experimental Raman spectra results.The morphologies were more significantly affected by extensional–shear coupled rates in the case of extensional–shear coupled flow, as well as shear rates in the case of simple shear flow and extensional rates in the case of simple extensional flow. Within the considered range of extensional–shearcoupled rates, the morphologies evolved from micelle-like structure to chain-like structure as the coupled rates increased from 0.01 to 2.0, showing a linear distribution along the flow direction. It was evident by the increase of MSD value upon increasing rates and an almost similar trend of relative concentration distribution while subjecting the system to the same rates in relation to different flow.

## Supplementary Materials

The following are available online at https://www.mdpi.com/2073-4360/11/8/1275/s1. Figure S1. The energy and pressure that reaches steady state as equilibrated.
