# Supplementary material for: A Dissipative Particle Dynamics Study of Flow Behaviors in Ultra High Molecular Weight Polyethylene/Polyamide 6 Blends Based on Souza-Martins Method
Source: Polymers (Basel). 2019 Jul 31;11(8):1275. doi: 10.3390/polym11081275 (PMC6722510; doi:10.3390/polym11081275)
Supplement: Supplementary file 1 [file polymers-11-01275-s001.pdf]

A dissipative particle dynamics study of flow behaviors in ultra high molecular weight polyethylene/polyamide 6 blends based on Souza-Martins method

Junxia Wang[a], Changlin Cao[a], Xiaochuan Chen[a], Shijie Ren[b], Dingshan Yu[a*]

a *Key Laboratory for Polymeric Composite and Functional Materials of Ministry of Education and Key Laboratory of High Performance Polymer-based Composites of Guangdong Province, School of Chemistry, Sun Yat-Sen University, Guangzhou510275, China;*

b *State Key Laboratory of Polymer Materials Engineering, Sichuan University, Chengdu610065, China*

* Corresponding Author E-mail: yudings@mail.sysu.edu.cn(D.S. Yu)

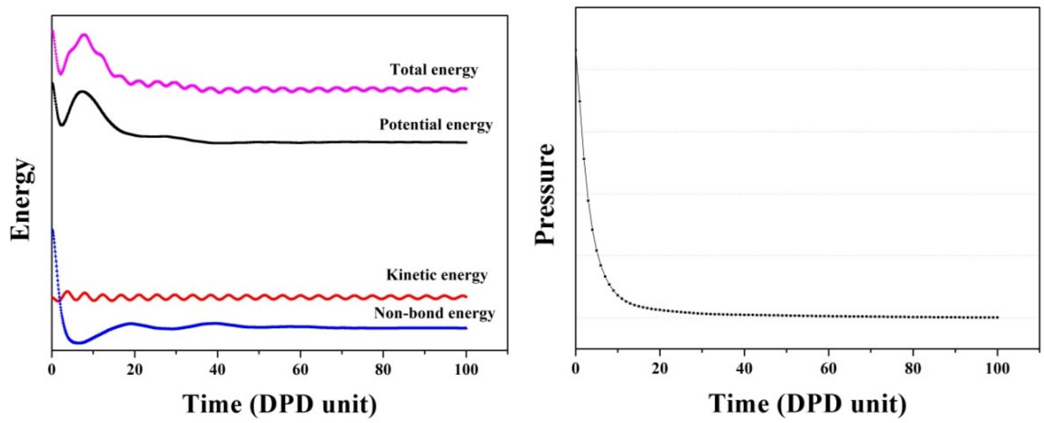

Figure S1 The energy and pressure that reaches steady state as equilibrated
